# Identification of Potential Therapeutic Targets in the Liver of Pioglitazone-Treated Type 2 Diabetes Sprague-Dawley Rats via Expression Profile Chip and iTRAQ Assay

**DOI:** 10.1155/2018/8120847

**Published:** 2018-03-18

**Authors:** Zhong-Xia Lu, Wen-Jun Xu, Yang-Sheng Wu, Chang-Yu Li, Yi-Tao Chen

**Affiliations:** ^1^College of Life Sciences, Zhejiang Chinese Medical University, Hangzhou 310053, China; ^2^College of Pharmacy, Zhejiang Chinese Medical University, Hangzhou 310053, China

## Abstract

The aim of the present study was to identify key antidiabetic nodes in the livers of pioglitazone-treated type 2 diabetes mellitus Sprague-Dawley rats by transcriptomic and proteomic analysis. Rats were randomly divided into the control, the diabetes model, and the pioglitazone-treated groups. After treatment with pioglitazone for 11 weeks, the effects on fasting blood glucose, body weight, and blood biochemistry parameters were evaluated. Microarray and iTRAQ analysis were used to determine the differentially expressed genes/proteins in rat livers. 1.5-fold changes in gene expression and 1.2-fold changes in protein were set as the screening criteria. After treatment with pioglitazone for 11 weeks, fasting blood glucose in pioglitazone-treated rats was significantly lower than that in the model group. There was a tendency for pioglitazone to reduce TC, TG, TP, ALB, BUN, and HDL-c levels. Kyoto Encyclopedia of Genes and Genomes (KEGG) and gene ontology (GO) were applied to analyze differentially expressed genes/proteins. Furthermore, Western blotting and RT-qPCR were used to validate the results of microarray and iTRAQ. In conclusion, *Cyp7a1*, *Cp*, and *RT1-EC2* are differentially expressed genes/proteins since they showed a similar trend in rats in the model group and the pioglitazone-treated group.

## 1. Introduction

Diabetes mellitus (DM) is a chronic metabolic disease affecting more than 400 million people worldwide [1]. Type 2 diabetes mellitus (T2DM) is caused by insulin resistance and/or insulin deficiency and constitutes almost 95 percent of all diabetes cases [2]. Previous studies have shown that several drugs, including metformin, rosiglitazone, and pioglitazone, can control high blood glucose levels and protect patients from diabetes-related complications [3].

Pioglitazone belongs to the thiazolidinedione class of drugs and was deemed as an insulin sensitizer for the therapy of T2DM [4]. As a highly selective agonist for peroxisome proliferator-activated receptor *γ* (PPAR-*γ*), pioglitazone regulates the transcription of several genes and proteins involved in glucose and lipid metabolism [5]. Shannon et al. reported that pioglitazone inhibited pyruvate-driven ATP synthesis and hepatocellular glucose production as well as suppressing mitochondrial pyruvate transport regulators, MPC1 and MPC2 [6]. Moreover, the expression of adiponectin is upregulated by pioglitazone [7]. Furthermore, the phosphorylation of AKT is decreased in T2DM mice while receiving pioglitazone treatment [8]. Therefore, it is obvious that pioglitazone is a multitarget and multipathway compound involved in antidiabetic efficacy. However, the key nodes involved in the multitarget antidiabetic effect of pioglitazone still need to be elucidated.

Network pharmacology, which is based on the concept that many drugs act on multiple rather than a single therapeutic target, is recognized as a kind of system biology and network analysis methodology and technology [9, 10]. Key molecular networks involved in biochemical networks, bioinformatics, and systems biology have been reconstructed, which are helpful in predicting novel targets with high efficiency [11–13]. For many drugs, including metformin, morin, and curcumin, several novel candidate targets were identified through network pharmacology assays [14–16].

In the present study, we established type 2 diabetes mellitus rats by injection of streptozotocin (STZ), and the potential therapeutic target nodes in the liver of pioglitazone-treated rats were screened by expression profile chip and iTRAQ assay. Furthermore, metabolic pathways regulated by pioglitazone were also explored.

## 2. Materials and Methods

### 2.1. Drugs and Reagents

Pioglitazone hydrochloride tablets were purchased from Takeda Pharmaceutical Industry (Osaka, Japan), rat GE 4x44K v3 microarrays and a Gene Expression Hybridization Kit were from Agilent Technologies (Shanghai, China), and QIAGEN RNeasy® Mini Kit was from QIAGEN (Shanghai, China). A prime Script RT Reagent Kit was from TaKaRa Biotechnology (Dalian, China).

### 2.2. Groups and Treatments

Male SD rats (three per cage, 200 g average body weight), 8 weeks of age, were obtained from the Animal Center of Zhejiang Chinese Medical University (Hangzhou, China). All animal experiments were carried out in Zhejiang Chinese Medical University Laboratory Animal Research Center (rodent license number SCXK 2013-0115). The experimental protocol was approved by the Ethics Committee of Zhejiang Chinese Medical University.

Before the experimental test, all animals were fed with the basic diet (Zhejiang Academy of Medical Sciences, Hangzhou, China) for 3 weeks. Subsequently, a type 2 diabetes model was established by injection of freshly prepared STZ (30 mg/kg) dissolved in ice-cold citrate buffer (0.1 M, pH 4.2). To obtain diabetic symptoms, T2DM rats were fed with high-glucose/high-fat diet, consisting of 69.75% basic diet, 10% lard, 10% yolk powder, 0.25% cholesterol, and 10% sucrose (Zhejiang Academy of Medical Sciences, Hangzhou, China), whereas 10 normal rats in the control group were fed with basic diet. Rats with blood glucose at 15–25 mmol/l were used in the experimental test, which were randomly divided into two groups, the diabetes model group (DM, *n* = 12) and the pioglitazone-treated group (n = 12). Rats in the control and the model group were given 0.25% CMC-Na (10 ml/kg, China National Pharmaceutical Group, Shanghai, China), whereas rats in the treated groups received pioglitazone (5 mg/kg, in 0.25% CMC-Na) via gavage once a day for 11 weeks. All animals were housed at 25°C with 50~70% humidity and a 12 h light/12 h dark cycle with free access to food and water.

### 2.3. Sample Collection and Index Detection

At the starting (0 week) and in the end (11th week) of the experiment, blood was collected from the orbital veins of the rats. The blood was used for the determination of biochemistry parameters, using commercially available kits in a semiauto analyzer (Photometer 5010 V5+, Berlin, Germany). Moreover, after an eight-hour fasting period, blood glucose levels were measured every two weeks using a glucose meter (Accu-Chek Performa, Roche Diagnostics GmbH, Mannheim, Germany). Body weights were recorded every two weeks. At the end of the experiment, rats were euthanized and livers were quickly dissected on ice. The livers were washed with DEPC water, frozen in liquid nitrogen, and stored at −80°C.

### 2.4. Microarray and iTRAQ Assay

A total of 18 samples (*n* = 6 samples per group) were used for microarray and iTRAQ assay. Total RNA was extracted from liver tissues using the MiniRNeasy Kit based on the manufacturer's instructions (QIAGEN, Shanghai, China). After the determination of the RNA integrity (Agilent Bioanalyzer 2100), RNA was hybridized to a Whole Rat Genome Oligo 4x44 K microarray (Agilent Technologies, Shanghai, China). The significantly differentially expressed genes were screened with a >1.5-fold differential expression, and *P* < 0.05 was considered statistically significant.

Total liver protein (100 *μ*g) from each sample was digested with trypsin, dried, and labeled with different tags per group. Control group samples labeled 114 tags, model group samples labeled 116, and pioglitazone-treated samples labeled 121. The labeled peptides were purified with a strong cation exchange chromatography (SCX) column (Phenomenex, California, USA) and separated by liquid chromatography (LC) using the LC-20AB HPLC Pump system (Shimadzu, Kyoto, Japan). Proteomic data collection was performed using a Triple TOF 5600 system (AB SCIEX, ON, USA) and a pulled quartz tip as the emitter (New Objectives, MA, USA). Proteins that were significantly different were screened with a >1.2-fold differential expression, and *P* < 0.05 cut-off in statistical analyses.

### 2.5. GO Enrichment and KEGG Analysis

The GO platform is a bioinformatics resource describing gene product attributes which have three categories, namely, biological process (BP), molecular function (MF), and cellular component (CC) [17]. We defined the significance of GO enrichment according to a *P* value, with a cut-off value of 0.05. The KEGG was applied to investigate if target genes were involved in multiple pathways. The top 10 pathway candidates that were potentially related to treatment were selected.

### 2.6. Reverse Transcription Quantitative Polymerase Chain Reaction (RT-qPCR) Assay

RT-qPCR was used to verify experimental data. Total RNA was extracted as described above, and double-strand cDNA was synthesized according to the manufacturer's instructions (TaKaRa Biotechnology, Shanghai, China). Primers were purchased from Sangon Biological Technology (Shanghai, China), of which the *Cyp7a1* former primer was 5′-GCATCTCAAGCAAACACCAT-3′, the downstream primer was 5′-TCCACTCACTTCTTCAGAGGC-3′, and the amplified fragment was 98 bp. Besides, the former primer of *Cp* was 5′-TGATGGCTATGGGCAATGA-3′, the downstream primer was 5′- GGTTTGGTATGTTCCAGGGA-3′, and the amplified fragment was 125 bp. Data were normalized to the expression level of a *β-actin* reference gene, in which upstream primer was 5′-GCTCTCTTCCAGCCTTCCTT-3′ and the downstream primer was 5′-GGTCTTTACGGATGTCAACG-3′. And its amplified fragment was 105 bp. RT-qPCR was performed with a Real Time PCR Machine (Eppendorf Realplex-4, Eppendorf, Germany).

### 2.7. Western Blotting Assay

Proteins (20 *μ*g) were separated by 12% SDS-PAGE and transferred onto PVDF membranes (Immobilon-FL membrane, Millipore Company, Massachusetts, USA). Then, the membranes were incubated with 5% milk in TBST (Tris-buffered saline and 0.5% Tween 20) and incubated overnight with antibodies against *Cyp7a1*, *Cp*, or *β-actin* (1 : 1000 Cell Signaling Technology, American) at 4°C, followed by incubation with goat anti-rabbit IgG HRP (Sangon Biological Technology, Shanghai, China) for 2 h. Finally, the membranes were visualized with a gel documentation system (Aplegen Omega Lum G, American).

### 2.8. Statistical Analysis

Data are expressed as the mean ± standard deviation (SD). SPSS software version 16.0 (Chicago, USA) was used for statistical analyses. Results of fasting blood glucose level, body weight, and biochemistry parameters were subjected to one-way analysis of variance (ANOVA). Western blotting results were quantitated by ImageJ software and analyzed by ANOVA. *P* < 0.05 was considered statistically significant.

## 3. Results

### 3.1. Effect of Pioglitazone on the Fasting Blood Glucose Level of Rats

**Figure 1:**
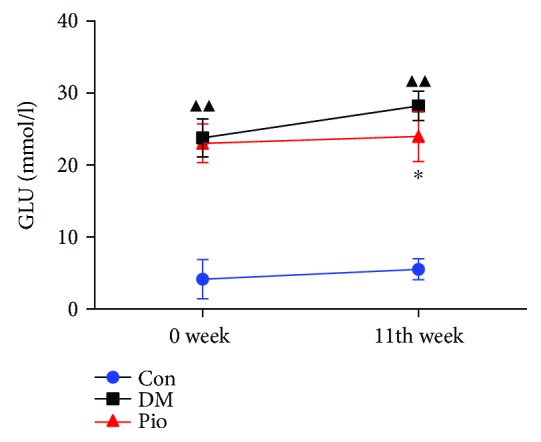
Pioglitazone reduces the fasting blood glucose level of DM rats. The fasting blood glucose levels in rats were significantly reduced after treatment with pioglitazone. ^▲▲^*P* < 0.01, the DM model group compared with the control group; ^∗^*P* < 0.05, the DM model group compared with the pioglitazone-treated group.

To examine the influence of pioglitazone *in vivo*, we administered pioglitazone to STZ-induced T2DM rats for a period of 11 weeks. As shown in Figure 1, the fasting blood glucose levels of DM rats and pioglitazone-treated rats were significantly higher compared to those of rats in the control groups at week 0 (*P* < 0.01). Moreover, after 11 weeks of treatment with pioglitazone, fasting blood glucose levels of pioglitazone-treated rats were significantly lower compared to those of rats in the DM model group (*P* < 0.05).

### 3.2. Effect of Pioglitazone on Body Weight of Rats

**Figure 2:**
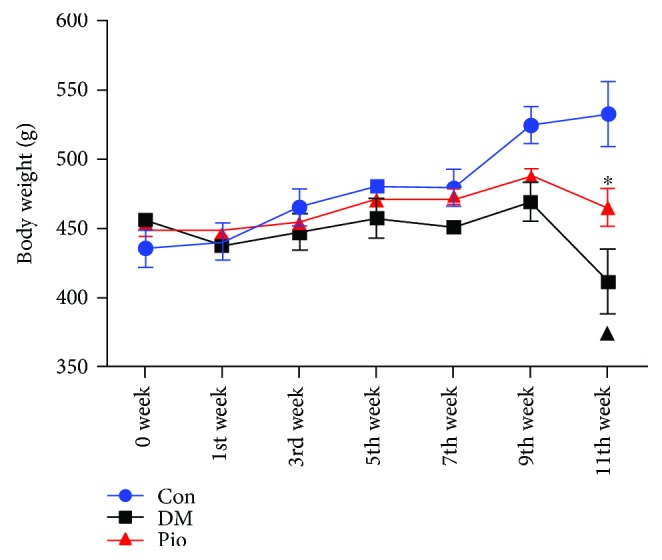
Pioglitazone alters the body weight of rats. The body weight of rats was recorded in 0, 1st, 3rd, 5th, 7th, 9th, and 11th weeks. ^▲^*P* < 0.05, the DM model group compared with the control group; ^∗^*P* < 0.05, the DM model group compared with the pioglitazone-treated group.

Body weight of rats in the control group, the DM model group, and the pioglitazone-treated group was also recorded in 0, 1st, 3rd, 5th, 7th, 9th, and 11th weeks. In the control group, the body weight of rats showed an upward trend from 435.83 ± 12.97 g in 0 week to 532.75 ± 23.18 g in the 11th week. However, rats in the DM model group demonstrated a significant loss in the body weight during the 11 weeks. Treatment with pioglitazone slightly increased the body weight of pioglitazone-treated rats and was significantly different compared with the body weights of rats in the DM model group (Figure 2, *P* < 0.05).

### 3.3. Effect of Pioglitazone on Biochemistry Parameters of Rats

**Figure 3:**
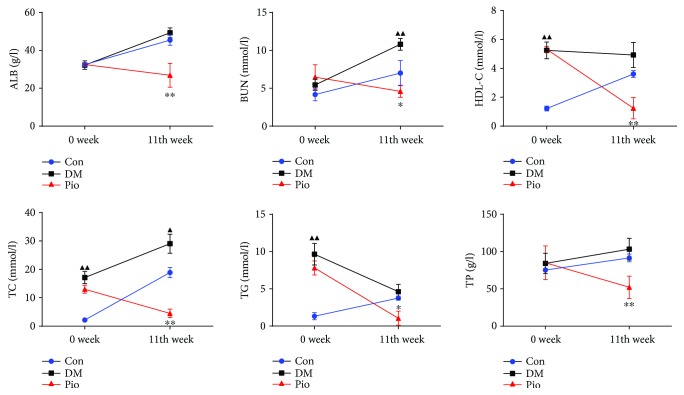
Pioglitazone changes blood biochemistry parameters. After treatment with pioglitazone for 11 weeks, the levels of TC, TG, TP, ALB, BUN, and HDL-c were significantly decreased. ^▲^*P* < 0.05; ^▲▲^*P* < 0.01, the DM model group compared with the control group; and ^∗^*P* < 0.05 and ^∗∗^*P* < 0.01, the DM model group compared with the pioglitazone-treated group.

Rats' plasma lipids were used for the evaluation of biochemistry parameters, including blood urea nitrogen (BUN), total cholesterol (TC), triglyceride (TG), high-density lipoprotein cholesterol (HDL-c), total protein (TP), and albumin (ALB). As shown in Figure 3, levels of the TC, TG, and HDL-c were significantly elevated in response to a high-glucose/high-fat diet (*P* < 0.01). After 11 weeks of pioglitazone treatment, TC, TG, TP, and HDL-c levels significantly decreased compared with rats in the DM model group (*P* < 0.05; *P* < 0.01). In previous studies, BUN and ALB measurements were commonly used to determine the liver function and renal function, respectively [18]. We found that levels of BUN and ALB were significantly decreased after pioglitazone treatment (*P* < 0.01), suggesting that pioglitazone has a protective effect on the kidney and liver.

### 3.4. Differentially Expressed Genes Screened by Expression Profile Chip

**Figure 4:**
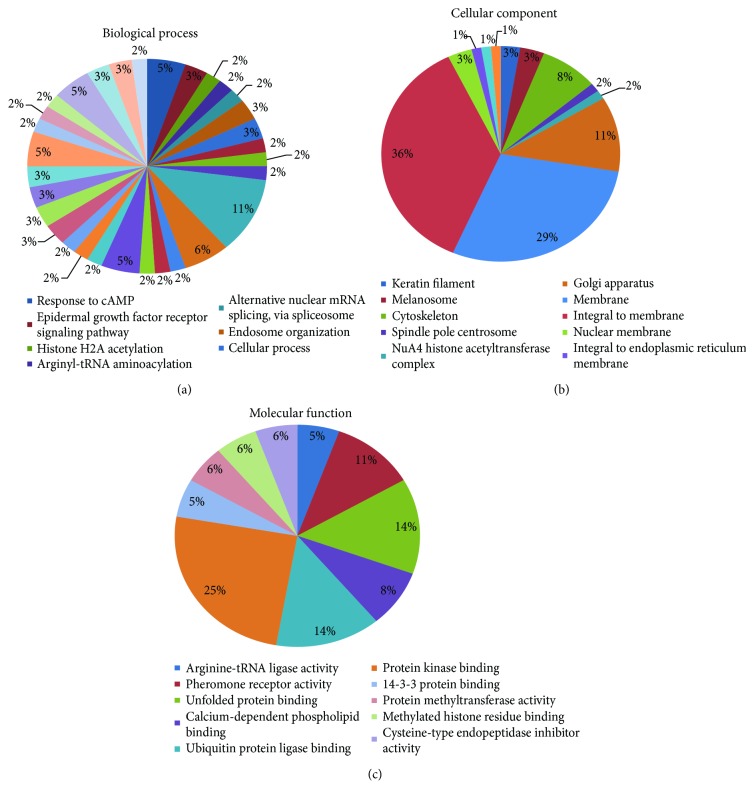
GO analysis of differentially expressed genes after treatment with pioglitazone. Microarray assay results of the DM model group and the pioglitazone-treated group. (a) Biological process. (b) Cellular component. (c) Molecular function.

By using a 1.5-fold change as the screening criteria for differentially expressed genes, a total of 322 genes were screened in the DM model group and the pioglitazone-treated group, in which 191 genes were significantly upregulated and 141 genes were significantly downregulated. GO enrichment and KEGG analysis were applied to assay the differentially expressed genes. As shown in Figure 4, pioglitazone treatment had a big impact on the biology process, the molecular function, and the cellular component in the liver. The biology process was affected by pioglitazone treatment, including the response to cAMP, epidermal growth factor receptor signaling pathway, and histone H2A acetylation. The pioglitazone-affected molecular function is involved for example in arginine-tRNA ligase activity, pheromone receptor activity, and unfolded protein binding. The cellular component was associated with keratin filament, melanosome, and cytoskeleton. As shown in Table 1, top 10 signaling pathways enriched by KEGG included ribosome biogenesis in eukaryotes, fatty acid metabolism, and protein processing in endoplasmic.

### 3.5. Differentially Expressed Proteins Screened by iTRAQ

**Figure 5:**
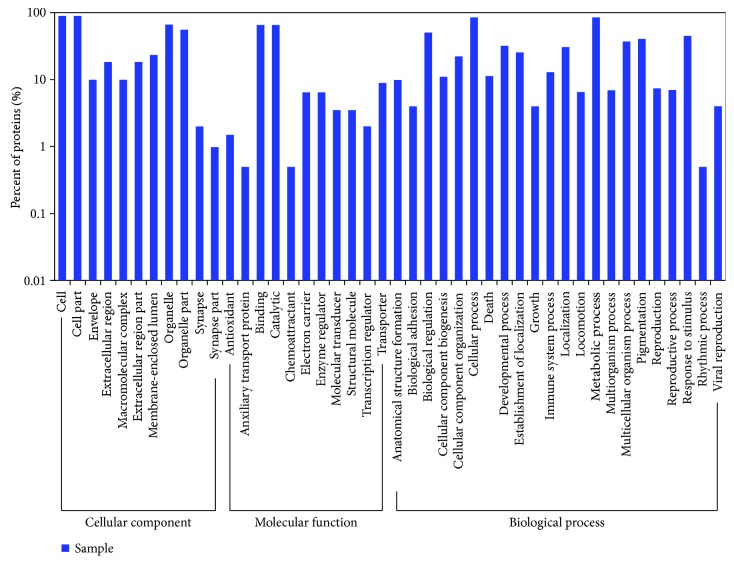
GO analysis of differentially expressed proteins after treatment with pioglitazone. iTRAQ assay results of the DM model group and the pioglitazone-treated group.

To explore the differentially expressed proteins in the liver, we applied iTRAQ to select potential targets with 1.2-fold change differences as the screening criteria. A total of 204 proteins were found as differentially expressed proteins in the DM model group and the pioglitazone-treated group, of which 96 proteins were significantly upregulated and 108 proteins were significantly downregulated. KEGG pathway enrichment results are presented in Table 2. Proteins with altered expression were categorized according to KEGG pathway analysis and included the retinol metabolism, steroid hormone biosynthesis, and metabolic pathways.  Figure 5 presents the gene ontology results. Cellular components affected by pioglitazone mainly included cellular parts, envelope, and extracellular regions. Using GO analysis, major molecular functions intervened by pioglitazone included antioxidant, auxiliary transport protein, and binding. Biological processes identified by GO assay of proteins are presented in Figure 5. The anatomical structure formation, biological adhesion, and biological regulation were among the main affected biological process.

### 3.6. Transcriptomic and Proteomic Conjoint Analysis of Potential Therapeutic Targets

Further analysis showed 3 molecules were found with the same trend in the pioglitazone-treated group after transcriptomic and proteomic conjoint analysis, namely, *Cyp7a1*, *Cp*, and *RT1-EC2.* Among these targets, the expression levels of *Cyp7a1* and *Cp* were significantly increased, whereas that of *RT1-EC2* decreased (Table 3). Therefore, *Cyp7a1*, *Cp*, and *RT1-EC2* were identified as potential therapeutic targets in the liver with pioglitazone treatment.

### 3.7. Validation of Potential Therapeutic Targets

**Figure 6:**
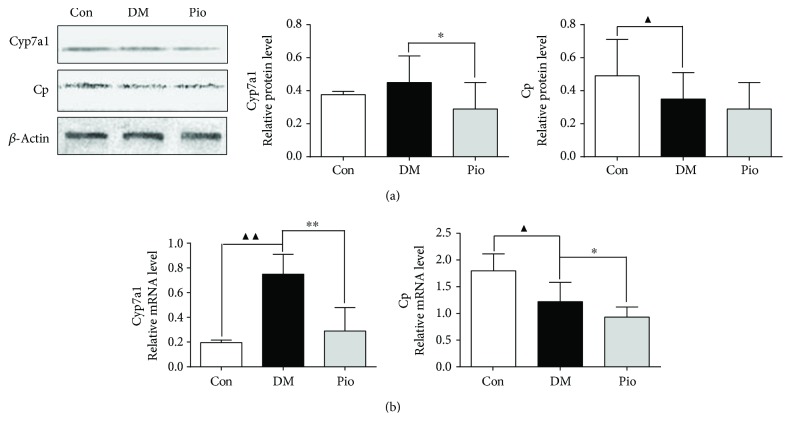
Data verified by RT-qPCR and Western blotting. (a) Gene expression detected by RT-qPCR. (b) Protein expression detected by Western blotting. ^▲^*P* < 0.05; ^▲▲^*P* < 0.01, the DM model group compared with the control group; and ^∗^*P* < 0.05 and ^∗∗^*P* < 0.01, the DM model group compared with the pioglitazone-treated group.

*Cyp7a1* (cytochrome P450, family 7, subfamily a, polypeptide I) and *Cp* (Ceruloplasmin) were further validated by qRT-PCR and Western blotting assay. As shown in Figure 6, compared with the DM model group, the gene expressions and protein levels of *Cyp7a1* in the pioglitazone-treated group were significantly decreased, while the gene expression level of *Cp* in the pioglitazone-treated group was also significantly downregulated after treatment with pioglitazone for 11 weeks. The results confirmed the former microarray and iTRAQ findings.

## 4. Discussion

T2DM is characterized by chronic hyperglycemia due to worsening insulin resistance, which is a hallmark of type 2 diabetes [19]. Practically, insulin resistance exists throughout the course of diabetes; thus, enhancing insulin sensitivity is a key strategy for treatment [20]. Pioglitazone is an antidiabetic drug for the clinical treatment of T2DM and has been used over the past 50 years. It is known to improve insulin sensitivity, glycaemic control, hypertension, dyslipidemia, and microalbuminuria acting on PPAR-*γ* [21, 22].

In the present study, we established T2DM model rats with STZ and a high-glucose/high-fat diet according to the Reed method [23]. Pioglitazone was given by gavage to treat T2DM rats. After treatment with pioglitazone, fasting blood glucose levels were significantly lower compared to that in the model group. In addition, the levels of TC, TG, TP, ALB, BUN, and HDL-c were also significantly reduced.

Microarray and iTRAQ, high-throughput bioinformatics technologies, are widely used in drug screening, new drug development, and disease diagnosis [24–26]. The liver is regarded as a metabolic center and plays vital important roles in material metabolism and energy metabolism by regulating the metabolism of numerous molecules, such as carbohydrates, lipids, proteins, and hormones [27]. Hence, in the present study, we selected the liver as the target organ to study the changes of genes and proteins after pioglitazone treatment. Based on the results from microarray and iTRAQ analysis, *Cyp7a1*, *Cp*, and *RT1-EC2* were selected as the most significantly differentially expressed targets.

Ceruloplasmin(*Cp*) is a ferroxidase enzyme synthesized in the liver and is involved in carrying copper in the blood and in addition plays a role in iron metabolism [28, 29]. *Cp* is also used as an index to assess hepatic disease; the expression level of *Cp* dropped due to reduced synthesizing capabilities after liver damage [30]. *RT1-EC2* is a class 1b gene of the rat major histocompatibility complex, which is reported to be responsible for the development of diabetes in rats [31, 32]. *Cyp7a1* belongs to the cytochrome P450 family of enzymes and encodes cholesterol 7a-hydroxylase, which is the rate-limiting enzyme in bile acid biosynthesis from cholesterol [33]. Previous studies have reported that *Cyp7a1* is associated with circulating cholesterol concentrations in response to various dietary interventions [34]. Furthermore, numerous studies have reported that *Cyp7a1* plays a vitally important role in modulation of bile acid, lipid, and glucose homeostasis [35–37]. Therefore, *Cyp7a1* may serve as a potential candidate therapeutic node for diabetes.

## 5. Conclusions

In summary, our study demonstrated that pioglitazone is a multitarget antidiabetic drug affecting numerous genes involved in lipid and cholesterol metabolism pathways. Through microarray and iTRAQ analyses, *Cyp7a1*, *Cp*, and *RT1-EC2* were the most differentially expressed molecules in the DM group and the pioglitazone-treated group. Moreover, our study provides valuable data that may help in elucidating the underlying mechanism of pioglitazone in T2DM.

## Figures and Tables

**Table 1:** KEGG pathway enrichment via gene expression profile chip (DM versus Pio).

Number	Pathway ID	Pathway	*P* value
1	ko03008	Ribosome biogenesis in eukaryotes	2.92*E* − 02
2	ko04977	Vitamin digestion and absorption	4.21*E* − 02
3	ko04141	Protein processing in endoplasmic reticulum	1.21*E* − 01
4	ko00051	Fructose and mannose metabolism	1.48*E* − 01
5	ko00790	Folate biosynthesis	1.62*E* − 01
6	ko00071	Fatty acid metabolism	1.69*E* − 01
7	ko00970	Aminoacyl-tRNA biosynthesis	1.69*E* − 01
8	ko04960	Aldosterone-regulated sodium reabsorption	1.69*E* − 01
9	ko03040	Spliceosome	1.71*E* − 01
10	ko04612	Antigen processing and presentation	1.85*E* − 01

**Table 2:** KEGG pathway enrichment via iTRAQ (DM versus Pio).

Number	Pathway ID	Pathway	*P* value
1	ko00830	Retinol metabolism	4.44*E* − 09
2	ko00140	Steroid hormone biosynthesis	2.49*E* − 06
3	ko01100	Metabolic pathways	8.68*E* − 06
4	ko00980	Metabolism of xenobiotics by cytochrome P450	1.54*E* − 05
5	ko00982	Drug metabolism—cytochrome P450	1.62*E* − 05
6	ko04976	Bile secretion	7.75*E* − 05
7	ko03320	PPAR signaling pathway	4.96*E* − 04
8	ko00590	Arachidonic acid metabolism	6.60*E* − 04
9	ko00920	Sulfur metabolism	2.41*E* − 03
10	ko00983	Drug metabolism—other enzymes	4.72*E* − 03

**Table 3:** Transcriptomic and proteomic conjoint analysis of potential targets.

Gene symbol	Transcriptomics		Proteomics		Regulation
	Log (Pio versus DM)	Log (Con versus DM)	Pio versus DM	Con versus DM	
*Cyp7a1*	−0.06	−1.52	0.488	0.361	Down
*Cp*	−0.17	−1.15	0.826	0.648	Down
*RT1-EC2*	0.21	0.24	1.255	1.204	Up

## Abbreviations

TC: Total cholesterol
TG: Triglyceride
TP: Total protein
ALB: Albumin
BUN: Blood urea nitrogen
HDL-c: High-density lipoprotein cholesterol
DM: Diabetes mellitus
T2DM: Type 2 diabetes mellitus
GO: Gene ontology
KEGG: Kyoto Encyclopedia of Genes and Genomes
Cyp7a1: Cytochrome P450, family 7, subfamily a, polypeptide I
Cp: Ceruloplasmin
RT1-EC2: RT1 class Ib, locus EC2.
